# Novel Approaches for Treating Autonomously Functioning Thyroid Nodules

**DOI:** 10.3389/fendo.2020.565371

**Published:** 2020-10-30

**Authors:** Pia Pace-Asciak, Jon O. Russell, Mohammad Shaear, Ralph P. Tufano

**Affiliations:** Division of Otolaryngology-Head and Neck Surgery, Johns Hopkins University, Baltimore, MD, United States

**Keywords:** benign thyroid nodules, toxic nodules, transoral endoscopic thyroidectomy, radiofrequency ablation, hyperthyroidism

## Abstract

Benign thyroid nodules are exceedingly common in the adult population. Only a small percentage of nodules are toxic or autonomously functioning thyroid nodules (AFTNs). The options clinicians have for treating the symptoms of hyperthyroidism include anti-thyroidal medications, radioactive iodine, or surgery. Depending on the patient population treated, these options may not be suitable or have inherent risks that are undesirable to the patient. On the other hand, untreated hyperthyroidism can lead to osteoporosis, atrial fibrillation, emotional lability, and neurological consequences. Thus, we present a review of two novel safe and effective approaches for treating AFTN; one surgical (transoral endoscopic thyroid surgery) and one non-surgical (radiofrequency ablation), as a means for expanding our treatment armamentarium.

## Introduction

Benign thyroid nodules (BTNs) are generally asymptomatic but can cause symptoms due to size or autonomous function. The frequency of incidental nodules is common in the adult population and can be found on high resolution ultrasound anywhere from 19 to 68%, with a higher proportion in females and the elderly (1). Over time, about 5–10% thyroid nodules can undergo progressive development to become autonomously functioning thyroid nodules (AFTNs) and secrete an abnormally higher amount of thyroid hormone. This may be more common in iodine deficient parts of the world (2, 3). AFTN is the second most common cause of hyperthyroidism, which occur more commonly in older women as these nodules degenerate with age (2).

Accompanying symptoms of hyperthyroidism can be overt or mild depending on the severity and include anxiety, emotional lability, weakness, tremor, palpitations, heat intolerance, increased perspiration, and weight loss despite a normal or increased appetite (4). Untreated hyperthyroidism can have deleterious effects on the cardiovascular and neurologic system, while also increasing the risk of osteoporosis and fractures (3, 4).

For decades, clinicians have treated the symptoms of hyperthyroidism with anti-thyroid drugs (ATDs) such as Methimazole or Propylthioruacil, Radioactive iodine ablation (RAI), or Surgery (4). Each treatment has unique risks, and the treatment therefore should be tailored to meet the needs of the patient population being treated. ATDs are commonly used to decrease thyroid hormone production and are useful in the short term, but they are not as definitive as surgery or radioactive iodine ablation (RAI) (3). Moreover, RAI may not be suitable in young women of child-bearing age, or patients who may not wish to endure the possibility of hypothyroidism (3). Surgery is a definitive intervention, but not all patients with AFTN are eligible for thyroid surgery or want surgery. While each of these strategies is appropriate for some patients, the inherent risks and limitations of each leave some patients seeking a more definitive option with more limited risks. In an age where there is a push towards ‘personalized medicine’, we review two recent advancements for the treatment of AFTN which may potentially broaden the surgical and non-surgical options for patients.

## Transoral Endoscopic Thyroid Surgery Vestibular Approach

For decades, surgical resection of the affected lobe or the whole thyroid using a mid-transcervical incision has been the primary surgical approach for AFTNs. Surgery is rarely the first option offered to patients in the Western Hemisphere (3). However, surgery is recognized as a safe and definitive option for the treatment of AFTN (3). Surgical removal of an AFTN is effective in alleviating the pressure from the trachea while also normalizing thyroid function. However, surgery is not without risk, and may be elevated in older patients (5). Potential surgical complications include the presence of a life-long visible scar despite having benign disease, recurrent laryngeal nerve injury, and temporary or permanent hypocalcemia (6, 7). Additionally, thyroid lobectomy has the 5–49% chance of leading to postoperative hypothyroidism (8–11).

Novel minimally invasive techniques have emerged over the years to avoid the need for a traditional mid-cervical scar. Transoral endoscopic thyroidectomy vestibular approach (TOETVA) has gained favor because it allows remote access to the thyroid through an incision in the mucosa of the lower lip, avoiding the need for any kind of visible scar in the neck (12). Other remote access techniques include the retroauricular approach, trans-axillary technique, and bilateral axillo-breast approach (13–16). To date, none of these other approaches have become common in the West, while TOETVA is becoming relatively popular due to broad operative indications, wide eligibility and applicability for patients with benign or malignant disease, and even with a BMI greater than 30 (17–19). Although TOETVA has longer operative time when compared to the conventional open technique, patient satisfaction and cosmesis are superior with TOETVA (20, 21).

TOETVA evolved from a sublingual incision to a small incision in the oral vestibule (12, 18). In the initial series of TOETVA, Anuwong et al. found TOETVA to be safe, efficacious, with quick recovery and a superior cosmetic outcome (12). The momentum for this novel approach gained favor among international circles, and several trials have reproduced the safety and efficacy of TOETVA in Asia, Europe, and the United States (18, 22–29). When compared to open surgical approaches, TOETVA has reliably demonstrated similar rates of postoperative infection, reduced pain, safety, and a similar complication profile to open techniques (recurrent laryngeal nerve injury and hypoparathyroidism) (17, 22, 25, 26, 30)

Surgical removal of AFTN is a recognized indication for TOETVA (31). Due to the novelty of this approach and low incidence of AFTN, the data regarding resection for AFTN is limited. There are no published studies specifically looking at the outcome of TOETVA in AFTN alone; rather toxic nodules are likely grouped into ’benign thyroid disease’ in the literature (23, 25, 32, 33) or TOETVA is compared to the open approach in patients with Graves disease (34). Given that one of the largest published series demonstrated a similar safety and complication rate for TOETVA compared to the traditional open approach, one can assume that the rate of success for treatment in AFTN would be similar to the open surgical open (22, 34). Luo et al. describe their case series of 204 patients; however, only one patient in their series demonstrated hyperthyroidism secondary to a secreting nodule as part of the ’benign nodule’ category (35). Future studies would need to address this aspect directly.

TOETVA has been shown to be a safe approach with minimal complications. Russell et al.’s analysis of literature yielded 689 cases whereby TOETVA outcomes were evaluated, and articles using robotic or floor of mouth techniques were excluded. Of the 689 cases that underwent TOETVA, there were no published cases of RLN injury or permanent hypoparathyroidism, and only one report of a hematoma (0.1%) (32, 36). Technique-specific complications, such as mental nerve injury and related hypoesthesia, CO2 embolism, and neck infection were extremely limited, with no cases of permanent mental nerve injury or CO2 embolism and only one case of neck infection (0.1%). Given that access is through an intraoral incision, perioperative antibiotic treatment is recommended to avoid potential infection. Furthermore, surgeons should be aware of the rare but potential complication of CO2 insufflation. CO2 embolism has been reported in four other cases where other transoral techniques were employed as well (two *via* a floor of mouth technique and two *via* the transoral vestibular approach) (37–39). Finally, 683/689 (99%) cases were completed *via* TOETVA without conversion to another technique. Five cases reported excessive bleeding that could not be controlled *via* TOETVA, and one case reported excessive tumor size with evidence of pretracheal nodal metastasis (32, 36).

Other smaller technique-specific complications are related to thinning of the skin or perforation of the skin flap in three cases (0.4%) resulting in a skin burn. In terms of postoperative pain control, some reports show TOETVA to be superior compared with the open thyroidectomy, but in our experience, pain control is relatively equal (22, 32, 34, 36).

For benign thyroid disease, the benefits of TOETVA may not be limited to cosmesis alone. From a surgical point of view, the birds-eye view magnifies the operative view and gives excellent visualization of the RLN and its insertion site. This allows for access to a favorable dissection plane centrally and bilaterally, providing a view that is familiar to most endocrine surgeons (25). The magnified view also enhances visualization of the parathyroid glands, allowing for their preservation and may reduce the risk of temporary postoperative hypocalcemia that can occur 14–40% of patients when accessing the central neck (40). Furthermore, even though the endoscopic approach affords a narrow working space, TOETVA provides a birds-eye view that enables the surgeon to have meticulous control of bleeding (12). While these theoretical benefits remain unproven, early evidence suggests that TOETVA is not inferior to the traditional surgical approach while offering improved cosmesis.

As with any new technique, there is a learning curve associated with implementing and refining the endoscopic approach which leads to longer operative times. Various authors have noted similar learning curves (22, 35, 41). As this technique becomes readily available at institutions, and surgeons become facile with this approach, it will likely become more available. There are very few barriers to adopting this technique since laparoscopic equipment is readily available at most institutions. Utilizing this novel approach adds very little extra cost making this approach accessible to motivated surgeons that wish to add TOETVA to their surgical armamentarium (42).

## Non-Surgical Approach: Radiofrequency Ablation

Radiofrequency ablation (RFA) is a novel minimally invasive approach that is a potential alternative to surgery for treating symptomatic benign nodules (43) as well as AFTN (44). This approach eliminates the need for a general anesthetic, an incision, radioactive iodine, or prolonged ATD treatment, making it an attractive non-surgical option. With the use of local anesthesia, the RFA probe is introduced into the midline of the anterior neck at the level of the isthmus, and the nodule is approached using the moving shot technique under ultrasound guidance (45). This causes tissue necrosis and fibrosis by introducing a high frequency alternating current, which raises tissue temperatures to 60 to 100 C (46). Over time, there is progressive shrinkage of the ablated nodule. In benign nodules, the volume of the nodule is thought to decrease between 50 and 80% for most patients, although this is operator and tumor dependent (47, 48).

RFA has been offered to patients internationally since 2000 and has been used to treat primary and metastatic tumors of the liver, lung, bone, and kidney and to ablate aberrant conduction pathways in the heart (49–53). More recently, RFA has been applied to the head and neck, particularly for thyroid nodules. While the early results have been promising internationally, there is little North American data (47, 48, 54–61). The current international recommendations for treating benign thyroid nodules include patients who are symptomatic or those who have a disfiguring goiter or a nodule that exceeds 2 cm, or if an AFTN is present (43, 44, 57). Prior to RFA treatment of AFTN, confirmation that the nodule is benign on at least one US-guided FNA or core biopsy is recommended, unless there are concerning features on ultrasound in which case two biopsies should be obtained (43, 44). Nodules that are benign on FNA but that have suspicious US features (EU-TIRADS 5), the latest European Thyroid Association Guidelines strongly recommend (moderate quality evidence) against thyroid ablation to avoid potential delay in treatment of a malignant lesion (62).

RFA has shown excellent efficacy and safety in the management of cosmesis related concerns and pressure symptoms (47, 48, 54–61, 63, 64). In a systematic review and meta-analysis of RFA in benign nodules, a pooled proportion of 2.38% for overall RFA complications was noted (95% CI: 1.42–3.34%), with 1.35% for major complications (95% CI: 0.89–1.81%) and no evidence of any life-threatening complications. The most common complaint post treatment was transient or rarely permanent voice changes (35/2,421). Nodule rupture, permanent hypothyroidism 6 months after treatment, and transient brachial plexus injury was only found in one patient out of 2,421 patients (65). Minor complications included pain during or after the procedure (16/2421), hematoma which disappeared after 1–2 weeks (25/2,421), vomiting (9/2,421), skin burns (six patients had first degree burns and 1 patient had second degree burn which recovered after a month) and transient thyroiditis (one patient three months after the treatment) (65). Furthermore, various studies have shown that the volume of a benign symptomatic thyroid nodule can be reduced by more than 50%, and up to 75–97% in long-term follow-ups (60, 66, 67).

The American Thyroid Association Guidelines outline that surgery or radioactive iodine (RAI) are effective for the treatment of AFTN (1, 3). These two options are not always acceptable for patients since RAI involves receiving radiation which is controversial in women of childbearing age, or for patients reluctant to endure the long-term risks associated with radiation (3). Additionally, both treatments have potential complications such as hypothyroidism. Even with lobectomy, surgery confers roughly a 30% chance of hypothyroidism, which is generally avoided in RFA-treated patients (10, 11, 68). RFA may gain favor with patients wishing to avoid developing hypothyroidism (55, 57, 58, 69–71).

Many trials have demonstrated the efficacy and safety of treating AFTN with RFA (55, 57, 58, 69–71). In a large multicenter trial, Sung JY et al. demonstrated improved symptoms of hyperthyroidism along with normalized TSH levels in 81.8% of study patients without the development of hypothyroidism post RFA (35, 69). In a systematic review, more than 50% of patients after RFA could discontinue their anti-hyperthyroid medications after RFA (44, 46). Additionally, patients that received RFA found significant improvement in their compressive symptoms due to the reduced nodule volume (mean volume reduction ratio, 81.7% during the mean follow-up period of 19.9 months). No major complications were reported in this trial; however, in previous trials, the most common complication reported was temporary pain (55, 57, 58, 63). Progression of hypothyroidism, if any, after treatment may be better explained by the progression of autoimmune thyroiditis associated with preexisting thyroid antibodies.

In a recent systematic review and meta-analysis, Cesareo et al. demonstrated moderate efficacy of RFA in AFTN (72). The overall rate of patients with TSH normalization or scintigraphically proven efficacy was about 60%, with a volume reduction of 79% found 1 year later in RFA treated AFTN (72). Bernardi et al. demonstrated that 50% of patients with AFTN treated with RFA withdrew their ATD after 12 months (70), which is similar to Deandrea (55) and Faggiano (73) who reported normalized thyroid function in 40% of patients after 12 months. However, Cervelli et al. demonstrated a greater volume reduction rate of 76% with a 91% TSH normalization at 12-month follow-up in AFTN treated with RFA (74). Even though there is heterogeneity between studies, and patients were only followed for 12 months, longer follow-up is warranted as well as how to achieve a predictable response rate with discontinuation of ATD after one session of RFA. It is also possible that these findings represent technical differences in the procedure, as it may be assumed that there is a learning curve with this procedure. Nevertheless, it is apparent that, while many patients benefit in the short term, a sizeable component may need additional treatment.

The success rate of RFA is greater when the volume of the AFTN is relatively small in size. Cesareo et al. compared the reduction between medium sized nodules (18 ml) and smaller sized nodules (5 ml), euthyroidism was achieved 86% in small nodules *vs.* 45% in medium size nodules (75). Similarly, Cappelli et al. report a volume reduction rate of 73% with TSH normalization in 94% of patients treated with RFA with nodules an average of 7 ml (76). An earlier study by Lim et al. confirmed that larger nodules (>20 ml) required repeat RFA treatment compared with smaller nodules to achieve a similar volume reduction in during 4 year follow-up (77). This work has improved our understanding of how to counsel patients with AFTN.

Since the Korean Guidelines in 2012, the consensus for RFA of AFTN has evolved. In line with the Korean Guidelines, the Italian Minimally Invasive Treatment of the Thyroid (MITT) group have advised clinicians to offer RFA as first line treatment in non-functioning nodules. However for AFTN, RFA is best reserved as second line treatment in patients who refuse conventional therapy or when it is contraindicated (75, 78). RFA can be considered as primary treatment for small sized AFTN (average 5 ml) since an optimal response (symptom improvement and TSH normalization) is noted when the nodule is reduced in size by more than 80% (72, 75).

Key factors that affect the therapeutic response of RFA on AFTN have been debated. Baek et al., demonstrated the importance of nodule vascularity on ultrasound for normalization in TSH and volume reduction (67), while Bernardi et al. found the percentage of volume reduction at 12 months correlated with the therapeutic response (70). Whereas, Cesareo et al.’s work demonstrates the importance of the size of nodule prior to RFA and the volume reduction after 12 months (75).

Localization of the nodule with ultrasound is also key in determining whether partial or complete ablation can be achieved, particularly if the nodule is adjacent to the trachea or recurrent laryngeal nerve. Ideally, complete ablation is preferred to avoid nodule regrowth. The moving shot technique is the standard approach that is used in conjunction with ultrasound for real time surveillance of nearby structures (43, 45). As with any awake technique, patient comfort is key in order to avoid patient movement which could result in injury to one of the neighboring structures. Light sedation can overcome this potential risk and ease patient comfort along with local anesthetic. Finally, the proficiency of the operator is important since there is a learning curve associated with complete removal of tissue to avoid recurrence of an untreated remnant. Tracing the residual viable area of the nodule by ultrasound (usually isoechoic and on the periphery of the ablation zone) can be helpful to predict regrowth and the area to target for reablation (79).

Single-session RFA has shown significant volume and symptom reduction (43, 45, 47, 48, 55–57, 66, 70, 72, 73, 76). Progressive reduction in the volume of treated nodules occurs incrementally over time, with reports ranging from 50 to 80% after 6 months and 79 to 90% after 2 years follow-up (55, 57, 70, 72). Previous studies report a mean number of RFA treatment sessions to be 1.8–2.2 (one to six sessions) for AFTN (58, 66). Previous reports show that single session RFA allowed withdrawal of ATD in 22–57% of patients (55, 70, 72), with one report showing 100% in pre-toxic nodules and 53% in toxic nodules (57). Other reports show a reduced dose of Methimazole after RFA in 78% of patients (55). The improvement in thyroid function is seen over time after RFA treatment, with up to 57% remission 12 months after the procedure (70, 72). However, other studies have demonstrated a tendency for regrowth after 2 to 3 years follow-up in non-functioning nodules (77, 79). The number of required treatments or how often nodules require RFA treatments still needs to be determined, especially when treating AFTNs. However, the pre-treatment nodule volume seems to play a key role in volume reduction and discontinuation of ATD. Currently, the Korean Guidelines suggest that follow-up should be based on TSH post RFA treatment of the AFTN. This will determine whether the patient’s antithyroidal medications can be stopped or if they require another treatment with RFA.

Imaging such as ultrasound examination and a thyroid scan with scintigraphy can also be helpful to know whether RFA was successful. Previous studies show post-treatment, scintigraphy demonstrated the majority of hot nodules (45–80%) became cold or normal, while 20.4–56% nodules had decreased uptake but still remained as hot nodules (58, 66). After treatment, evaluation *via* ultrasound should be done to ensure the absence of potential early complications from the procedure, such as an evolving hematoma, burns or damage to the thyroid capsule (62). On ultrasound, changes in the size, volume, intranodular vascularity and echogenicity are assessed (43). On ultrasound, the ablated area appears as a mildly hypoechoic and inhomogenous zone, with scattered hyperechoic spots due to tissue vaporization compared with the non-treated tissue (43, 62, 80). Color Doppler mapping of the treated areas are devoid of vascular signals (62, 80). If a patient is still symptomatic, the nodule can be assessed for persistent vascularity as a potential source for regrowth (81). The wide range of therapeutic effects of RFA may be attributed to remaining thyroid tissue left at the margin of the nodule. The best modality for detecting intranodular vascularity is contrast-enhanced ultrasound since color-Doppler is not as sensitive to detect small vessels and slow blood flow (43). Repeat RFA can be considered after 3 months from the first treatment because the most change is seen within the first 3 months (43, 69).

Given the potential for long-term cardiovascular mortality in the setting of untreated hyperthyroidism, the need for multiple treatments for patients treated with RFA does pose a theoretical risk that must be discussed appropriately (3, 57, 58, 70, 82). As newer evidence emerges, hopefully RFA will become standard of care. However, currently the Korean guidelines as well as the Italian MITT group discussed above take a more cautious tone when discussing the role of RFA in managing AFTN (43).

## Conclusion

AFTN are benign nodules with multiple treatment options. TOETVA and RFA are two novel treatments that may be safe and effective in the treatment of symptomatic benign thyroid nodules and could be applied to AFTN. TOETVA offers a surgical approach with the removal of the thyroid gland which avoids a cervical scar and is definitive, whereas RFA offers a non-surgical approach for patients who wish to avoid surgery altogether or are poor surgical candidates. Because there is risk associated with multiple rounds of treatment, it may not be appropriate for all patients and international guidelines suggest exercising caution (43). Each of these approaches may be appropriate in the right clinical setting.

## Author Contributions

PP-A reviewed the literature and wrote the paper. JR was involved with the writing and editing as well as the key concepts behind the paper. MS was involved with reviewing/editing the paper. RT was involved with editing as well as the key concepts behind the paper. All authors contributed to the article and approved the submitted version.

## Conflict of Interest

The authors declare that the research was conducted in the absence of any commercial or financial relationships that could be construed as a potential conflict of interest.
